# Rapid Raman spectroscopy-based test for antimicrobial resistance

**DOI:** 10.1098/rsob.240258

**Published:** 2025-02-26

**Authors:** Vladimir Mushenkov, Ksenia Zhigalova, Pavel Denisov, Alexey Gordeev, Dmitry Lukyanov, Vladimir Kukushkin, Tatiana Priputnevich, Elena Zavyalova

**Affiliations:** ^1^ Chemistry Department, Lomonosov Moscow State University, Moscow, Russia; ^2^ National Medical Research Center for Obstetrics, Gynecology and Perinatology named after Academician V.I. Kulakov of Ministry of Healthcare of Russian Federation, Moscow, Russia; ^3^ Center for Molecular and Cellular Biology, Skolkovo Institute of Science and Technology, Moscow, Russia; ^4^ Osipyan Institute of Solid State Physics Russian Academy of Science, Chernogolovka, Russia

**Keywords:** antibiotics, antimicrobial resistance, MTT, Raman spectroscopy

## Abstract

Antimicrobial resistance (AMR) is one of the top global health threats. In 2019, AMR was associated with 4.95 million deaths, of which 1.97 million were caused by drug-resistant infections directly. The main subset of AMR is antibiotic resistance, that is, the resistance of bacteria to antibiotic treatment. Traditional and most commonly used antibiotic susceptibility tests are based on the detection of bacterial growth and its inhibition in the presence of an antimicrobial. These tests typically take over 1–2 days to perform, so empirical therapy schemes are often administered before proper testing. Rapid tests for AMR are necessary to optimize the treatment of bacterial infection. Here, we combine the MTT test with Raman spectroscopy to provide a 1.5 h long test for minimal inhibitory concentration determination. Several *Escherichia coli* and *Klebsiella pneumoniae* strains were tested with three types of antibiotics, including ampicillin from penicillin family, kanamycin from aminoglycoside family and levofloxacin from fluoroquinolone family. The test provided the same minimal inhibitory concentrations as traditional Etest confirming its robustness.

**Figure:**
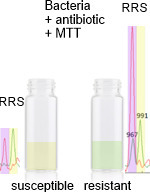

## 1. Introduction

Antimicrobial resistance (AMR) is one of the top global health threats. In 2019, AMR was associated with 4.95 million deaths, of which 1.97 million were caused by drug-resistant infections directly [1]. The main subset of AMR is antibiotic resistance, that is, resistance of bacteria to antibiotic treatment. Antibiotic resistance threat escalates quickly with widespread, and often incorrect, use of antibiotics [2]. The significant decline in new antibiotic discoveries exacerbates the problem even more. Most antibiotics were discovered in 1950−1970s, in the ‘Golden Age of antibiotics’, and since 1987, no new class of antibiotics has been discovered for use as treatment [3,4].

In the United States alone, AMR increased healthcare cost by $20 billion, and it is estimated to cause $35 billion loss in productivity annually [5]. Drug-resistant infections are difficult to treat, resulting in more expensive therapy and longer hospital stays. Even if therapy is administered, there is a significant rate of its inefficiency, with therapy failure rates as high as 54% for MRSA infections [6].

In drug-resistant infection treatment, a fast therapy appointment is crucial. For life-threatening conditions, such as sepsis, appropriate therapy needs to be applied within the first hours after diagnosis [7]. Traditional and most commonly used antibiotic susceptibility tests (ASTs) are based on the detection of bacterial growth and its inhibition in the presence of an antimicrobial [8]. These ASTs typically take over 1−2 days to perform, so empirical therapy schemes are often administered before the results of ASTs [9]. Because of the delay, AST results are often not used at all [10]. The delayed appropriate antibiotic therapy can result in prolonged hospital length of stay and higher mortality rate [11].

### 1.1. MTT assay for assessing bacterial cell viability

MTT assay is based on the enzymatic reduction of tetrazolium salts, commonly MTT (3-(4,5-dimethylthiazol-2-yl)-2,5-diphenyltetrazolium bromide), to violet insoluble formazan [12]. In living eukaryotic cells, MTT can be reduced by oxidoreductase and dehydrogenase enzymes, mainly associated with electron transport chain in mitochondria [12]. The reaction rate depends on cell metabolic activity; therefore, only living and active cells will sustain efficient MTT reduction. The amount of formazan formed correlates with the cell metabolism level and cell viability in the drug-containing media [13]. MTT reaction is applied for cell proliferation estimation [14,15] and cytotoxicity evaluation [16,17]. Various anticancer drug screenings rely on the MTT test to detect tumour cell suppression [18,19].

While the MTT assay was originally designed as an assay for eukaryotic cells, this method was also used for microbial cell viability estimation [20]. Although the mechanism of MTT reduction in bacteria is not understood well, there are numerous reports which apply MTT assay for bacteria, either Gram-positive or Gram-negative, including multidrug-resistance strain identification [21,22], antibiotic minimal inhibitory concentration (MIC) evaluation [23,24], detection of biofilm formation [25] and neutrophilic bactericidal activity evaluation [26]. The MTT assay was applied for estimating the number of growing cells of *M. tuberculosis* strains for rapid evaluation of drug resistance. In this case, the MTT assay allowed the test to to performed within 5−7 days, instead of 3−4 weeks for the standard colony counting method [27].

### 1.2. Raman spectroscopy

Raman spectroscopy is a label-free, noninvasive technique, which is based on nonelastic light scattering [28]. Raman scattered light has a shift specific to vibrational states of a molecule, and a Raman spectrum is a unique fingerprint of a substance [29]. Relatively low efficiency of inelastic scattering could be a limitation to sensitivity of analyses, so special modification was developed to overcome low light intensity—surface-enhanced Raman spectroscopy (SERS) and resonance Raman spectroscopy (RRS) [30].

SERS intensifies the signal due to the surface plasmon resonance effect. This effect occurs on rough metal surfaces, such as surfaces of nanoparticles, the most commonly used SERS-active substrate [31]. Signal is enhanced up to 10^14^ times, which brings SERS to single molecule level of detection [32]. However, signal amplification highly depends on the properties of the SERS-active surface; and the production of reproducible substrates is a rather complex task [33].

RRS occurs when the excitation light wavelength is close to the absorption maximum of a molecule and light frequency could become in resonance with the frequency of molecular electronic transitions [34]. In addition to signal enhancing (up to 10^6^ times), in a resonance Raman spectrum additional bands emerge, which refers to transitions undetectable by standard Raman scattering, such as overtones and combinational modes; therefore, additional information can be obtained from the spectrum [35].

### 1.3. A combination of resonance Raman spectroscopy and MTT assay

Although spectrophotometry in the visible range of the spectrum is a common practice for formazan detection in MTT assay, it has some disadvantages. First of all, formazan absorbance results are sensitive to the media, so an extraction step is necessary. Even after the extraction of formazan from the sample, formazan spectrum could be interfered with by protein precipitation caused by organic solvents [36]. Spectrophotometric assay lacks sensitivity taking several hours for the assay.

Raman spectroscopy provides a characteristic spectrum of formazan that can be used for a specific determination of a compound in a complex media. Characteristic formazan spectral lines are not interfered with by other substances in the sample, so steps of extraction and purification of formazan are not required. Sensitivity of Raman spectroscopy detection of formazan is comparable with that of spectrophotometry. However, the sensitivity of Raman scattering could be increased substantially with use of surface-enhanced or resonance Raman approaches [37,38].

The intensity and the Raman shift of characteristic peaks of formazan and MTT depend on the excitation light wavelength. Optimal wavelengths for MTT and formazan spectral measurements are 532 and 633 nm, respectively. A 633 nm laser provides a resonance Raman spectrum of formazan [39]. Comparable sensitivities for both MTT and formazan could be obtained using an excitation wavelength of 532 nm. Several peaks in the formazan spectrum were shown to have a linear dependence on formazan concentration, being suitable for a quantitative analysis. Namely, a peak at 967 cm^−1^ for 532 nm excitation wavelength and peaks at 722 and 967 cm^−1^ for 633 nm excitation wavelength are valuable for a quantitative analysis [40].

In this study, we developed a rapid AST that is based on a combination of RRS and MTT assay. The method has turnaround time of about 1.5 h (disregarding time needed for cell growth for samples with low concentration) and has low labour intensity. We used the method on antibiotic-resistant and susceptible strains of *Escherichia coli* and *Klebsiella pneumoniae* to estimate MICs. The estimated MICs were compared with MICs obtained by a common technique, broth dilution.

## 2. Material and methods

### 2.1. Reagents

MTT bromine salt was obtained from Dia-M (Russia). MTT was dissolved in water, diluted to a concentration of 4 mg ml^−1^ and stored at 4°C for a week. LB broth (Lennox) was purchased from Condalab (Italy). Antibiotics (levofloxacin, kanamycin and ampicillin) were purchased from Sigma-Aldrich (USA). Phosphate buffer solution (PBS) with pH 7.2 contained 0.8% NaCl, 0.02% KH_2_PO_4_, 0.02% KCl and 0.12% Na_2_HPO_4_·12H_2_O. In this study, MilliQ water was used. All chemicals were analytical grade reagents.

### 2.2. Bacterial cultures

ΔtolC, ΔtolC-GyrA-D87Y [41] and ΔtolC-KanR (JW5503) [42] strains of *E. coli* were obtained from the own collection of the laboratory of Olga Dontsova. LB liquid medium was prepared from LB broth (Lennox) via the manufacturer’s protocol. An amount of 4 g of the solid medium was suspended in 200 ml of MilliQ water, mixed and sterilized in an autoclave for 15 min at 121°C. Prepared medium was stored at 4°C. The bacterial strains were inoculated onto agar dishes at 37°C; then a single colony was grown in LB medium at 37°C overnight.

Clinical samples (*K. pneumoniae* 0739_23, *K. pneumoniae* H934_23, *E. coli* W879_23, *E. coli* A1832_23, *E. coli* w3237_23 and *E. coli* w3239_23) were taken from the working collection of Institute of Microbiology, Antimicrobial Therapy and Epidemiology of National Medical Research Center for Obstetrics, Gynecology and Perinatology named after Academician V.I. Kulakov of Ministry of Healthcare of Russian Federation. The collection is a part of the research work ‘Studying the mechanisms of resistance in clinically significant microorganisms to antimicrobial drugs with the formation of a collection of strains of microorganisms and the development of a test system containing molecular markers new mechanisms of resistance of opportunistic microorganisms’; the study was approved by the Ethics Committee of the National Medical Research Center for Obstetrics, Gynecology and Perinatology named after Academician V.I. Kulakov of Ministry of Healthcare of Russian Federation (Protocol No. 9, 23 September 2021).

The *E. coli* strain W879_23 was isolated from the vaginal discharge of a pregnant woman (21 weeks of pregnancy) with a tumour of the uterine body requiring medical care to the mother. The strain was resistant to ampicillin and amoxicillin/clavulanic acid; and it was sensitive to gentamicin, cefepime, ceftriaxone, imipenem and levofloxacin.

The *E. coli* strain W3237_23 was isolated from the vaginal discharge of a pregnant woman (35 weeks of pregnancy) with threatening premature birth, placental insufficiency, grade 1 obesity, insulin therapy for gestational diabetes mellitus. The strain was resistant to ampicillin, amoxicillin/clavulanic acid, cefotaxime, gentamicin and ciprofloxacin; it was sensitive to cefepime, meropenem, ertapenem, amikacin and colistin.

The *E. coli* strain W3293_23 was isolated from the urine of a pregnant woman (39 weeks of pregnancy) with oedema of pregnant women and latent iron deficiency. The strain was sensitive to ampicillin, amoxicillin/clavulanic acid, cefepime, ceftriaxone, imipenem, gentamicin and levofloxacin.

The *E. coli* strain A1832_23 was isolated from the mucous membrane of the cervical canal of a pregnant woman (37 weeks of pregnancy) with urolithiasis, multiple uterine fibroids and autoimmune thyroiditis. The strain was resistant to ampicillin, amoxicillin/clavulanic acid, cefepime, ceftriaxone, gentamicin and levofloxacin; and it was sensitive to imipenem.

The *K. pneumoniae* strain H934_23 was isolated from a rectal smear of a newborn child with jejunum atresia type II and incomplete bowel rotation. The strain was resistant to ampicillin, amoxicillin/clavulanic acid, cefotaxime, ceftazidime, imipenem, meropenem, gentamicin and amikacin.

The *K. pneumoniae* strain O739_23 was isolated from the oropharyngeal and nasopharyngeal mucosa of a newborn child with multiple malformations including congenital heart disease: common arterial trunk type 2. The strain was resistant to ampicillin, amoxicillin/clavulanic acid, cefotaxime, ceftazidime, imipenem, meropenem, gentamicin and amikacin.

Cultures of clinical strains were grown during 12−18 h on a non-selective agar culture medium Columbia Agar Base (Thermo Scientific Oxoid, UK) at 37°C. Columbia Agar Base was prepared in accordance with the manufacturer’s instructions. An amount of 39 g of agar was diluted in 1 l of distilled water, boiled until the medium was completely dissolved, sterilized by autoclaving at 121°C for 15 min, cooled to 50°C and 5% sterile defibrinated blood was added. The prepared dishes with the nutrient medium were stored at a temperature of 2–8°C.

For the preparation of dilutions during the MTT assay, Mueller–Hinton broth (Thermo Scientific Oxoid, UK) was used. The Muller–Hinton broth was prepared in accordance with the manufacturer’s instructions. An amount of 21 g of Muller–Hinton broth was suspended in 1 l of distilled water until completely dissolved and sterilized in an autoclave at a temperature of 121°C for 15 min. The prepared Muller–Hinton broth was stored at a temperature of 2–8°C.

### 2.3. Etest

Muller–Hinton agar (Thermo Scientific Oxoid, UK) was prepared in accordance with the manufacturer’s instructions. An amount of 38 g of agar was added to 1 l of distilled water. This was brought to a boil to completely dissolve the nutrient medium. The medium was sterilized by autoclaving at 121°C for 15 min. The prepared dishes were stored at a temperature of 2–8°C. A narrow strip of polymer (0.5 × 6.0 cm^2^) with a gradient of antibacterial drug concentrations was used. The strip was placed on the surface of a Muller–Hinton agar dish with a bacterial culture from a standard dilution (0.5 MFU). Immediately after applying the test strips, the Petri dishes were placed upside down in a thermostat and incubated at a temperature of 35°C for 18 ± 2 h. After incubation, the result was read using a dilution scale applied to the strip at the intersection of the growth retardation zone in the form of an ellipse.

### 2.4. Instruments and measurement parameters

In this study, two Raman spectrometers were used. Spectrometers were bought from Enspectr (Russia) and Photon-Bio (Russia). 532 nm wavelength Raman spectra were obtained using the Enspectr RaPort spectrometer, and 637 nm spectra were obtained using the Photon-Bio RL637 spectrometer.

Optical density measurements were made with an ECROS 5400UV spectrophotometer (Russia).

Raman spectra for both wavelengths were collected with exposure time set at 1300 ms for 20 repeats. Laser powers for 532 nm and 637 nm excitation were set at 30 and 100 mW, respectively.

Collected spectra were smoothed by the Whittaker filtering algorithm [43] and baselines were corrected with asymmetrically reweighted penalized least squares smoothing [44].

### 2.5. Raman-assisted MTT antibiotic resistance assay

Bacterial strains were obtained from overnight cultures, diluted with media to 2 MFU (McFarland Units, around 6 × 10^8^ cells ml^−1^), cell concentration was measured using a spectrophotometer at 570 nm (OD_570_ for 2 MFU = 0.451) and incubated at 37°C for an hour. Then 50 μl of cell culture was added to 350 μl of PBS with the required amount of antibiotic and incubated at 37°C for another 1 h. After the incubation, 100 μl of MTT was added to the samples, and after incubation for 20 min, samples with a total volume of 500 μl were placed into glass vials and measured with a spectrometer.

Intensity of formazan peak at 967 cm^−1^ was collected from each spectrum from samples with antibiotic and related to those of the control sample without antibiotic. The final intensity values were presented as a percentage of the intensity of the control sample.

## 3. Results and discussion

### 3.1. Raman spectra of MTT, formazan and bacteria after MTT treatment

In this work, portable Raman spectrometers with lasers of 532 and 637 nm wavelengths were used. The spectrometers were equipped with holders for 2 ml sealed vials. The devices allow investigation of liquid biological samples with no cross-contamination inside the spectrometers.

**Figure 1.**
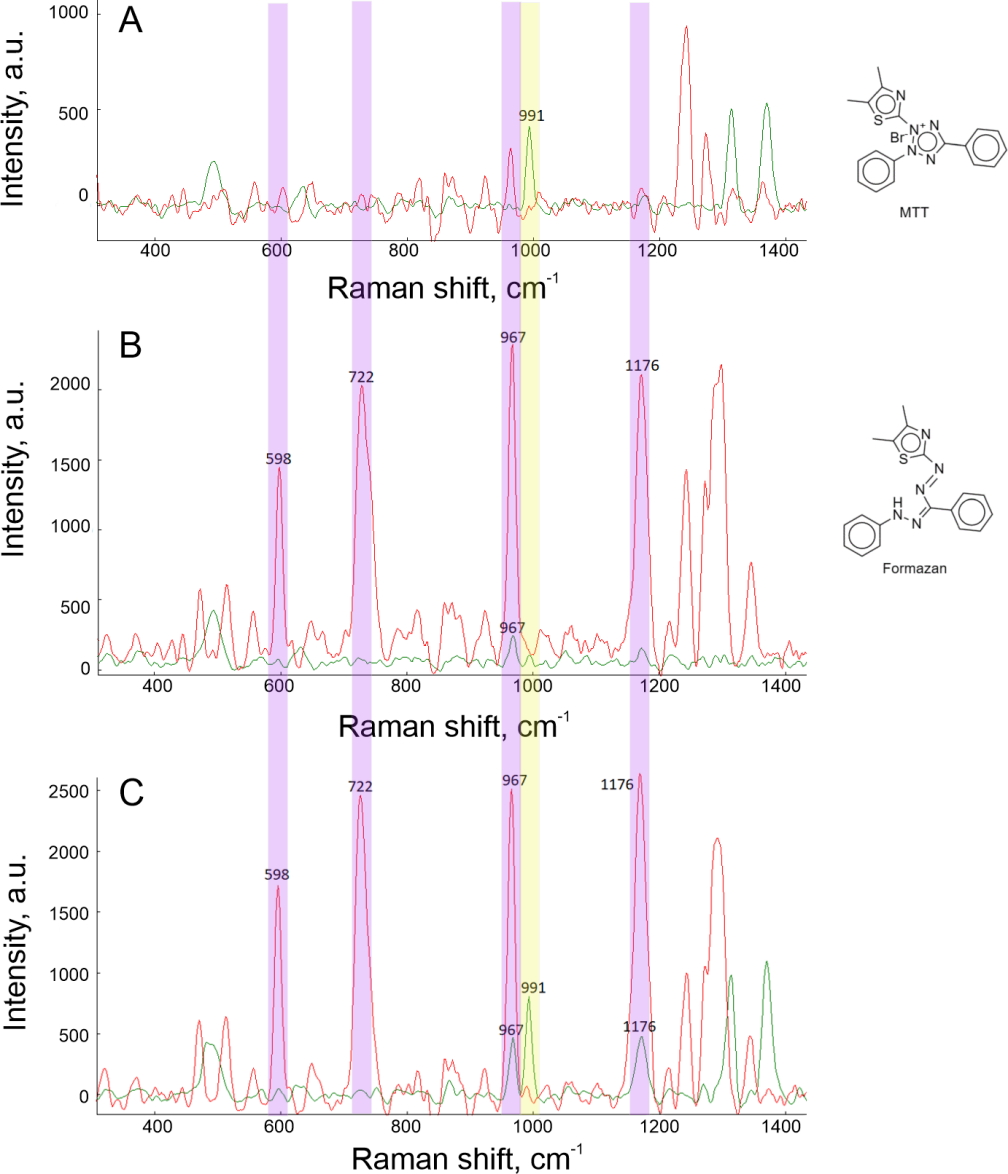
Raman spectra of 0.4 g l^−1^ MTT solution in water (A), 0.02 g l^−1^ formazan suspension in water (B), and *E. coli* after incubation with MTT reagent (C). Spectra were collected with 532 and 637 nm excitation wavelengths of Raman spectrometer (green and red colours, respectively). Characteristic bands are assigned: MTT band is in yellow; formazan bands are in purple. The chemical formulae of MTT reagent and formazan are shown in insets of (A,B), respectively.

First, we compared the suitability of these spectrometers for formazan detection in the suspension of living bacterial cells. Figure 1 shows the comparison of Raman spectra of MTT and formazan at the two excitation wavelengths. MTT and formazan have unique Raman spectra that allow discrimination of these two substances in mixtures. The 532 and 637 nm excitation wavelengths provided nearly equal intensity for MTT. However, intensities of formazan spectra differed by an order of magnitude being much higher with 637 nm excitation wavelength. This observation is consistent with a previous study that reported the resonance Raman spectrum of formazan at 633 nm excitation wavelength of the Raman spectrometer [39].

Raman spectroscopy allows decoding of a mixture of MTT, formazan and living cells in the culture medium. The representative spectra are shown in figure 1. *E. coli* bacterial culture (2 MFU) was incubated with MTT for 30 min. Spectra of bacterial culture have characteristic bands for both compounds. Spectra with 532 nm excitation wavelength indicate nearly half-conversion of MTT into formazan. In the case of 637 nm excitation wavelength, the MTT signal is barely visible, while the formazan signal is high due to the resonance effect. Both excitation wavelengths can be used to estimate MTT conversion in formazan; but 637 nm excitation wavelength was considered to provide higher sensitivity due to the enhancement supported by the resonance effect.

**Figure 2.**
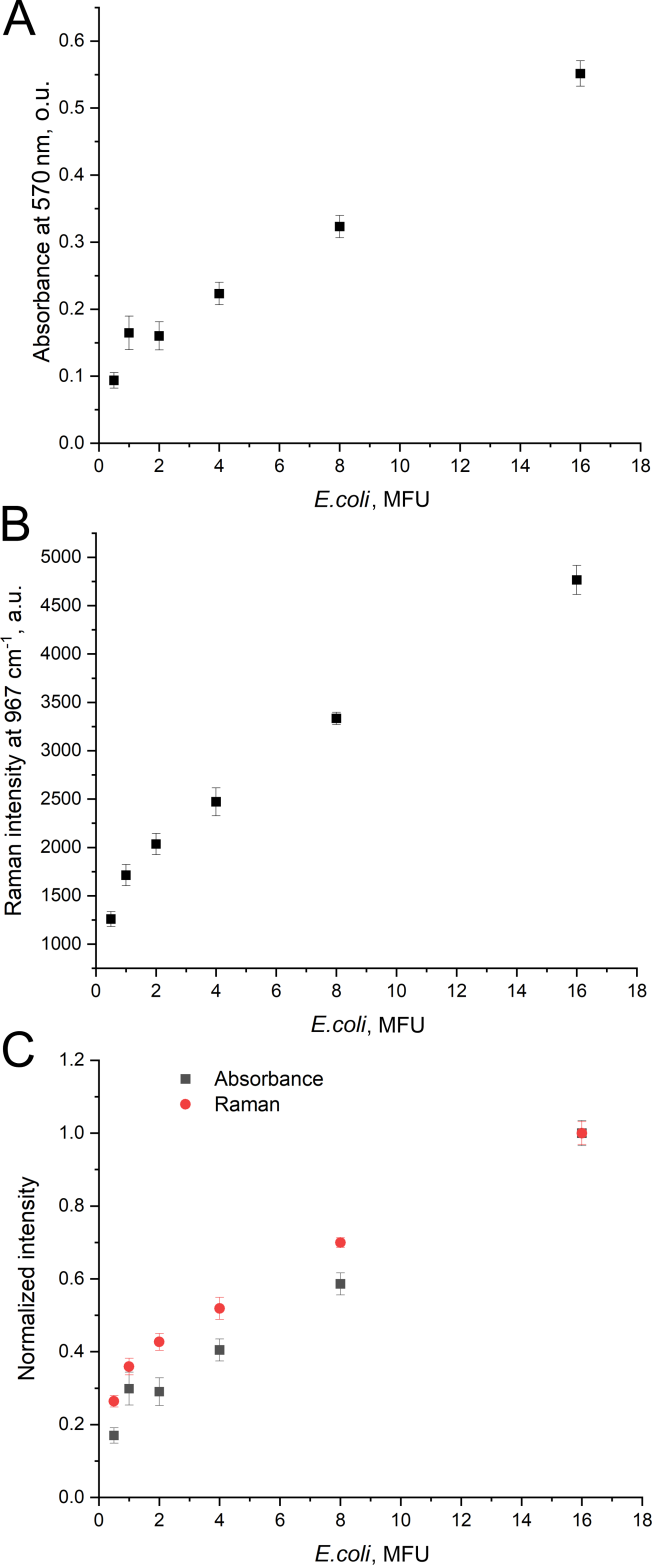
A comparison of optical density of MTT-treated *E. coli* samples (A) and Raman spectra intensity of MTT-treated *E. coli* (B) in different bacterial titre. Normalized values were acquired by dividing the values from (A) and (B) by the values for 16 MFU samples (C).

### 3.2. Development of the Raman spectroscopy-based antimicrobial resistance test

First, we have found that night cultures in stationary growth phase provide a poor RRS signal. The cultures were diluted with a fresh medium and incubated for 1 h at 37°C. The RRS signal increased more than 5 times as a result. Changes in the visible range of the spectrum are commonly used to estimate the quantity of bacteria in cultures [45,46]. The increase in cell quantity during the elaborated AMR test was assessed with optical density at 600 nm. We found no statistically significant changes in bacterial titre for the samples with 0.5−5 MFU. Possibly, *E. coli* were in the lag phase during 1−2 h for the rapid test performance.

Next, formazan accumulation was determined with absorption and Raman spectroscopies. The wavelength of 570 nm was used for formazan detection in the visible range. The optical density changes nearly linearly; however, estimation of the samples with low bacterial concentrations (<2 MFU) was not linear. Possible reasons include the increase of light scattering contribution and rather large standard deviations compared to the absolute values (figure 2A). Raman spectroscopy provided a smooth monotonic dependence in the whole concentration range studied with a linear range from 2 to 16 MFU (figure 2B). Raman spectroscopy provided the specific determination of formazan instead of the composite signal in absorption spectroscopy.

RRS is a more precise technique for low content of bacteria. Normalized spectra (figure 2C) are rather close to each other. RRS provided nearly the same results as absorption spectroscopy emphasizing the relationship between the quantity of the bacteria and their enzymatic activity in MTT test.

**Figure 3.**
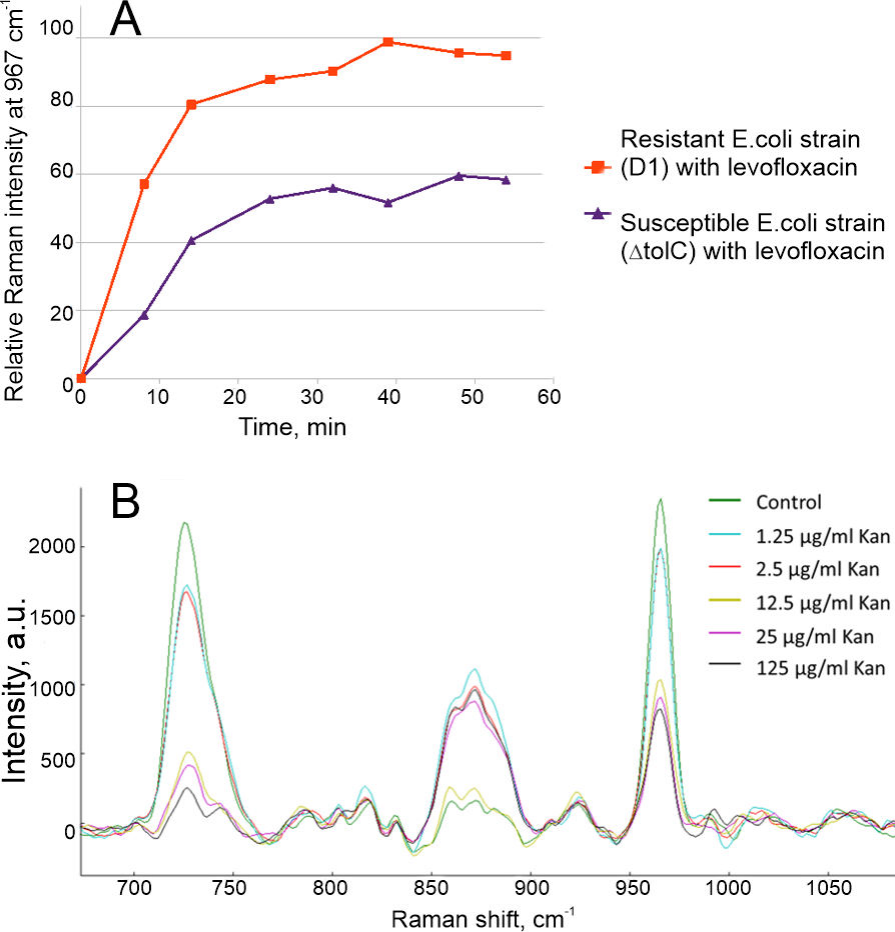
Time dependence of Raman intensity for levofloxacin-resistant (ΔtolC-GyrA-D87Y) and susceptible (ΔtolC) *E. coli* strains in the presence of 13 µg ml^−1^ of levofloxacin (A). Raman spectra of kanamycin-susceptible strain of *E. coli* (ΔtolC-GyrA-D87Y) after treatment with different concentrations of kanamycin (B).

Next, the antibiotic susceptibility assay was conducted. Levofloxacin-resistant and susceptible *E. coli* strains were treated with 13 µg ml^−1^ of levofloxacin for 1 h at 37°C. Then, the MTT reagent was added to the probe, and formazan accumulation in the probe was studied with RRS. The levofloxacin-resistant *E. coli* strain gave a plateau of formazan RRS intensity after 30 min of incubation. Similarly, levofloxacin-susceptible *E. coli* strain reached the plateau of the signal after 30 min of incubation; but the intensity of the signal was nearly 2 times lower compared to the resistant strain (figure 3A). Possibly, the formazan signal was not diminished completely as cells were not killed but exerted toxic effects from the antibiotic. The time of incubation with MTT was fixed at 20 min for further experiments in order to reach a plateau.

The concentration dependence for another antibiotic is shown in figure 3B. *E. coli* ΔtolC-GyrA-D87Y strain was treated with kanamycin in different concentrations. Increasing concentration of the drug leads to a decrease in the formazan signal intensity. The signal changed abruptly from nearly 100% to 50% at 2.5 µg ml^−1^. This value might be due to residual metabolic activity of some enzymes or by some reducing agents [47–49].

### 3.3. Performance of the antimicrobial resistance test on the antibiotic-resistant and susceptible *E. coli* strains

**Figure 4.**
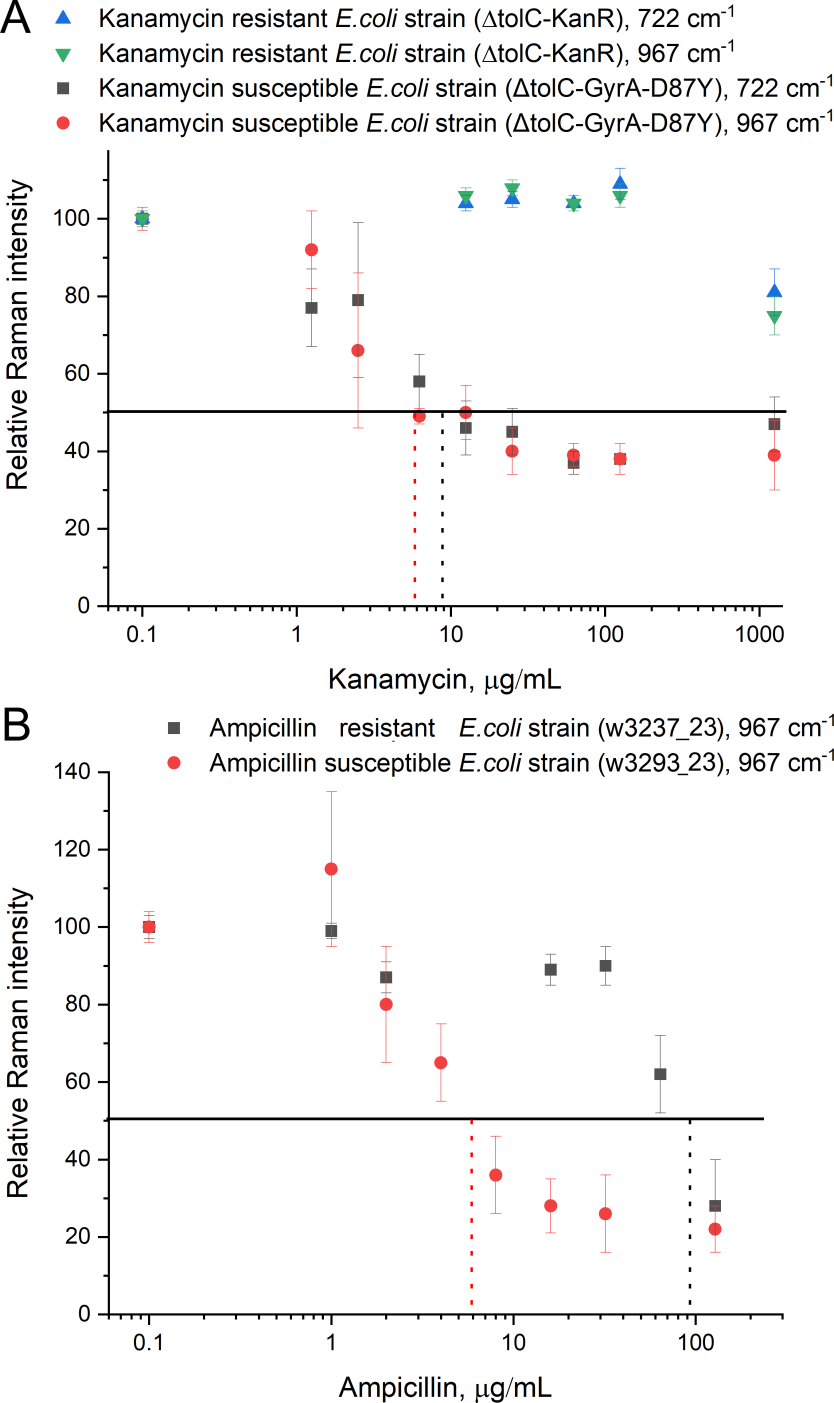
AMR test on the kanamycin-susceptible and resistant *E. coli* strains (A) and ampicillin-susceptible and resistant *E. coli* strains (B). 50% of Raman intensity was used as a reference value for antibiotic susceptibility.

Two pairs of antibiotic-susceptible and resistant *E. coli* strains were chosen to test the performance of the novel AMR test. Kanamycin-resistant (ΔtolC-KanR) and susceptible (ΔtolC*-*GyrA-D87Y) *E. coli* strains were compared (figure 4A). The intensities of two formazan bands were followed in both samples with varying concentration of kanamycin.

The Raman intensities were normalized to the values from the sample without the antibiotic. The most intense Raman bands of formazan, 722 and 967 cm^−1^, were compared. Both bands provided similar concentration dependencies (figure 4A), so any of these bands can be used in the AMR test. MIC can be estimated by taking 50% of Raman intensity as a reference value of susceptibility to the antibiotic. The MICs were 6 and 9 µg ml^−1^ for 967 and 722 cm^−1^ dependencies, respectively, for the kanamycin-susceptible *E. coli* strain (ΔtolC-GyrA-D87Y). These values are in good agreement with MIC of 5 µg ml^−1^ that was determined with Etest [50]. The kanamycin-resistant *E. coli* strain (ΔtolC-KanR) had MIC larger than 1000 µg ml^−1^.

Similar experiment was conducted with ampicillin-resistant (w3237_23) and ampicillin-susceptible (w3239_23) clinical strains of *E. coli* (figure 4B). The 967 cm^−1^ band was chosen for evaluation. Again, at some concentration signal decreases rapidly, and further ampicillin concentration increase affects intensity slightly. Both strains had ampicillin-dependent decreases in Raman signal, but MICs differ by 15 times, namely 6 µg ml^−1^ for susceptible strain and 90 µg ml^−1^ for resistant strain.

### 3.4. Direct comparison with conventional techniques in minimal inhibitory concentration determination

For this experiment, several clinical strains of *E. coli* and *K. pneumoniae* were chosen that are resistant or susceptible to levofloxacin. MICs of levofloxacin for these strains were established with a conventional AMR technique (Etest). Simultaneously, MICs were determined with RRS (a novel AMR test) using 50% decrease in Raman signal as a reference value. The results are provided in table 1. The MICs determined with these two techniques were the same, demonstrating good performance of the novel test.

**Table 1.** Comparison of MICs to levofloxacin determined with the novel AMR test (resonance Raman spectroscopy, RRS) and standard AMR (Etest). (S) means susceptible strain, (R) means resistant strain. The relative standard deviations varied from 5% to 10%.

levofloxacin (μg ml^−1^)	*K. pneumoniae* 0739_23 (S)	*K. pneumoniae* H934_23 (R)	*E. coli* W879_23 (S)	*E. coli* A1832_23 (R)
0	100	100	100	100
0.5	94	100	53	100
2	88	97	54	100
4	50	96	50	99
8	48	92	60	95
64	43	91	47	96
256	55	45	57	83
MIC (RRS)	4	>64	0.5	>64
MIC (Etest)	4	>32	0.5	>32
comparison	essential agreement	essential agreement	essential agreement	essential agreement

The RRS method was compared with Etest as described in [51]. According to these criteria, essential agreement is defined when new and reference methods have similar MIC values, and categorical agreement is defined when the results of both methods were interpreted in the same category (resistant or susceptible). All RRS method results were in essential agreement with Etest results. However, the number of samples is insufficient to ensure statistical significance of these results. Large-scale studies are necessary.

The developed AMR test provided a rapid determination of MIC during 1.5 h for a fresh bacterial culture. The MTT test is known to be applicable to a wide variety of bacteria. RRS provides a possibility of independent specific determination of formazan without destroying the cells with detergents. The method provided reliable MIC values for six bacterial isolates of *E. coli* and *K. pneumoniae* and three types of antibiotics, including ampicillin from penicillin family, kanamycin from aminoglycoside family and levofloxacin from the fluoroquinolone family.

One of the most popular techniques for determining antibiotic sensitivity is the disc diffusion test; it remains the most common in practical bacteriological laboratories to date. In addition, the sequential dilution method in either agar or broth is commonly used for determining MIC in laboratory practice. Also, the gradient diffusion method (Etest) is rather popular. All these techniques take about 24 h for unfastidious microorganisms (e.g. 18 h for *E. coli*) and much longer for fastidious ones. MTT assay coupled with Raman spectroscopy provided similar results in just 1.5 h providing a possibility of a rapid AMR test.

## Data Availability

This article has no additional data.
